# Retraction Note: Discovery of nanoscale sanal flow choking in cardiovascular system: exact prediction of the 3D boundary-layer-blockage factor in nanotubes

**DOI:** 10.1038/s41598-023-36260-8

**Published:** 2023-06-06

**Authors:** V. R. Sanal Kumar, Vigneshwaran Sankar, Nichith Chandrasekaran, Sulthan Ariff Rahman Mohamed Rafic, Ajith Sukumaran, Pradeep Kumar Radhakrishnan, Shiv Kumar Choudhary

**Affiliations:** 1 Indian Space Research Organisation, VSSC, Trivandrum, Kerala 695022 India; 2 Indian Institute of Science, Bangalore, Karnataka 560012 India; 3 Kumaraguru College of Technology, Coimbatore, Tamil Nadu 641049 India; 4 Indian Institute of Technology, Kanpur, Uttar Pradesh 208016 India; 5 GITAM University, Visakhapatnam, Andhra Pradesh 530045 India; 6 All India Institute of Medical Sciences, New Delhi, 110608 India

Retraction of: *Scientific Reports* 10.1038/s41598-021-94450-8, published online 29 July 2021

The Editors have retracted this Article.

Following publication, concerns were raised about the rationale for the approach presented, the assumptions and approximations used and the validity of its application to cardiology. A post-publication review of the Authors' mathematical arguments revealed a lack of clarity in the terms presented and inferences that are not adequately justified. The main concerns are that the model is based on circular reasoning which makes it non-predictive, that it assumes that blood behaves as an ideal gas, and hypothesizes that quasi-sonic flow velocities exist in the cardiovascular system while all experimental evidence shows that cardiovascular flow velocities are orders of magnitude lower than the speed of sound and do not involve any compressibility effects. The Editors therefore no longer have confidence in the conclusions presented.

None of the Authors has responded to correspondence from the Editors about this retraction.

